# Atomic Sulfur Passivation Improves the Photoelectrochemical Performance of ZnSe Nanorods

**DOI:** 10.3390/nano10061081

**Published:** 2020-05-31

**Authors:** Fei Huang, Jiajia Ning, Wei Xiong, Ting Shen, Yanling Zhao, Jianjun Tian, Ruiqin Zhang, Andrey L. Rogach

**Affiliations:** 1 Institute for Advanced Materials and Technology, University of Science and Technology Beijing, Beijing 100083, China; ting_shen@sutd.edu.sg (T.S.); tianjianjun@mater.ustb.edu.cn (J.T.); 2 Department of Physics, City University of Hong Kong, Hong Kong 999077, China; weixiong4-c@my.cityu.edu.hk (W.X.); apzyl@cityu.edu.hk (Y.Z.); aprqz@cityu.edu.hk (R.Z.); 3 Center for Functional Photonics (CFP), City University of Hong Kong, Hong Kong 999077, China; jiajning@cityu.edu.hk (J.N.); andrey.rogach@cityu.edu.hk (A.L.R.); 4 Department of Material Science and Engineering, City University of Hong Kong, Hong Kong 999077, China

**Keywords:** photoelectrochemical cell, heavy metal-free photocatalyst, ZnSe nanorods, atomic sulfur passivation, ZnS monolayer

## Abstract

We introduced atomic sulfur passivation to tune the surface sites of heavy metal-free ZnSe nanorods, with a Zn^2+^-rich termination surface, which are initially capped with organic ligands and under-coordinated with Se. The S^2−^ ions from a sodium sulfide solution were used to partially substitute a 3-mercaptopropionic acid ligand, and to combine with under-coordinated Zn termination atoms to form a ZnS monolayer on the ZnSe surface. This treatment removed the surface traps from the ZnSe nanorods, and passivated defects formed during the previous ligand exchange process, without sacrificing the efficient hole transfer. As a result, without using any co-catalysts, the atomic sulfur passivation increased the photocurrent density of TiO_2_/ZnSe photoanodes from 273 to 325 μA/cm^2^. Notably, without using any sacrificial agents, the photocurrent density for sulfur-passivated TiO_2_/ZnSe nanorod-based photoanodes remained at almost 100% of its initial value after 300 s of continuous operation, while for the post-deposited ZnS passivation layer, or those based on ZnSe/ZnS core–shell nanorods, it declined by 28% and 25%, respectively. This work highlights the advantages of the proper passivation of II-VI semiconductor nanocrystals as an efficient approach to tackle the efficient charge transfer and stability of photoelectrochemical cells based thereon.

## 1. Introduction

It is ever important to establish efficient photocatalytic water splitting systems, in order to obtain clean and easily storable hydrogen fuels for solving energy and environment issues [1,2]. Compared with semiconductor powder-based photocatalytic water splitting systems, photoelectrochemical (PEC) cells can effectively suppress the backward reactions, thus enabling us to collect hydrogen more easily [3,4,5]. The photocatalyst is at the core of PEC cells, and thus extensive efforts have been devoted to the development of abundant, inexpensive, nontoxic, and efficient photocatalysts [6,7,8,9,10,11,12]. Among those, colloidal semiconductor nanocrystals (NCs), with a physical size smaller than the exciton Bohr radius, offer some useful properties as compared with bulk semiconductors [13,14,15,16,17]. Their valence and conduction band positions, and thus the redox potentials of photogenerated holes and electrons, can be adjusted on demand for a given material while tuning the particle size, due to the quantum confinement effect. The large surface area of the colloidal nanoparticles provides abundant surface reaction sites and promotes their contact with electron donors and/or acceptors in PEC cells. At the same time, this leads to a high ratio of under-coordinated surface termination atoms, which are usually passivated with organic capping ligands, mostly long-chain alkyl amines, or alkyl acids in the case of NCs synthesized in organic solvents [18,19,20,21,22]. Those termination atoms may lead to the formation of deep or shallow mid-gap states, which often increases the chance for nonradiative or thermal exciton dissipation [23,24,25,26]. For the colloidal semiconductor NCs to be applied in PEC, in order to promote charge transfer and facilitate their efficient loading on metal oxide substrates, their original long-chain alkyl-based ligands are commonly exchanged by short chain bifunctional ligands, such as thioglycolic acid (TGA) or 3-mercaptopropionic acid (3-MPA) [27]. However, this ligand exchange process can easily introduce additional surface traps, causing inefficient charge carrier transfer, which then in turn results in the degradation of the anode materials and the overall PEC performance [28,29,30]. We note that it is also possible to employ II-VI NCs, which are directly synthesized in water, employing short ligands TGA or MPA, in order to achieve more efficient charge transfer at the interface [31,32,33]. However, the aqueous-based TGA or MPA-capped NCs still face a high ratio of surface termination atoms. Tailoring the surface states of semiconductor NCs is thus particularly important for improving the performance of PEC cells. One common method is the growth of a shell of a wider bandgap material on the NC surface to construct the so-called core–shell structures [29,34,35]. In the core–shell heterostructures, surface defects are normally situated at the outer surface of the shell, thus lowering the probability of trapping photogenerated charge carriers, which are generated in the core by the surface site. However, an improper shell thickness would hinder the charge carrier transport in core–shell NCs, while the lattice mismatch between the core and the shell materials may introduce defects at the core–shell interface, which would compromise the optoelectronic properties [36]. Another frequently applied method is the deposition of the metal chalcogenide (ZnSe or ZnSe) on an NC-based photoelectrode via a successive ionic layer adsorption and reaction (SILAR) or chemical bath deposition (CBD) [37,38]. This approach was rather successful in improving photocurrent density due to the reduced amount of surface defects.

Other than the Cd- or Pb-based NCs, which contain heavy metals [39,40], ZnSe is a II-VI semiconductor material with a direct bandgap of 2.7 eV and suitable conduction band edge position located at around −1.1 V vs. NHE (pH = 0), which has been regarded as a promising heavy metal-free photocatalyst for water splitting [17,41]. In view of the superior charge carrier separation in elongated nanorods (NRs), which can enhance PEC performance, we applied ZnSe NRs and suggested an atomic sulfur passivation method to passivate the surface sites on ZnSe nanorods (NRs) and to improve the performance of the respective PEC cells, which was accomplished by dipping ZnSe NR-based photoanodes into a sodium sulfide solution. The Zn/Se atomic ratio in the initial ZnSe NRs has been determined to be 1.4:1 by inductively coupled plasma–atomic emission spectrometry (ICP–AES), meaning that they possess a Zn^2+^-rich termination surface. X-ray photoelectron spectroscopy (XPS) analysis indicated that upon the treatment with sodium sulfide, S^2−^ ions substituted a large fraction of surface ligands and combined with the Zn termination atoms to form a ZnS monolayer. Without using any sacrificial reagents and/or co-catalysts, this atomic sulfur passivation method increased the photocurrent density of a TiO_2_/ZnSe NR photoanode from 273 μA cm^−2^ to 325 μA cm^−2^ as compared with the same photoanode without passivation. Furthermore, after atomic sulfur passivation, the photoanode showed no obvious decline in photocurrent density after 300 s of continuous operation, while the photoanodes with a post-deposited ZnS passivation layer, or those based on ZnSe/ZnS core–shell NCs, exhibited photocurrent density decays of 28% and 25%, respectively. Photoelectrochemical characterization has shown that an improved charge transfer and a suppressed charge recombination was achieved with the aid of S^2−^ passivation.

## 2. Experimental Section

### 2.1. Chemicals

Zinc acetate dihydrate (>99%), selenium (powder, 99.5%, 100mesh), 1-dodecanethiol (1-DDT, 98.0%), 1-octadecene (ODE, 90%), and hexane (99%, anhydrous) were purchased from Sigma-Aldrich (St. Louis, MO, USA). Oleylamine (OLA, approximate C18 content 80−90%) was purchased from Arcos (NY, USA). 3-mercaptopropionic acid (3-MPA, >90%, Sigma Aldrich), sodium hydroxide (NaOH, 98%, Sigma Aldrich), titanium oxide (TiO_2_, Degussa, Corporation, Essen, Germany, P25), α−terpineol (C_10_H_8_O, 96%, Sigma Aldrich), ethyl cellulose ([C_6_H_7_O_2_(OC_2_H_5_)_3_]_n_, 48.0−49.5% (w/w) as ethoxyl, Sigma Aldrich), zinc acetate dihydrate (Zn(AC)_2_·2H_2_O, 98.0%, Alfa Aesar, Haverhill, MA, USA), sodium sulfate (Na_2_SO_4_, 98.0%, Alfa Aesar), hydrochloric acid (HCl, 37%, Sigama Aldrich), methanol (CH_3_OH, ≥99.5%, Sigma Aldrich), and ethanol (CH_3_CH_2_OH, ≥99.5%, Decon Labs, King of Prussia, PA, USA) were directly used without further purification. Ultrapure deionized water was used for the preparation of all aqueous solutions.

### 2.2. Synthesis of ZnSe NRs

The synthesis was carried out using standard airless techniques on a vacuum/dry argon gas Schleck line system. Zinc acetate dihydrate (219.51 mg, 1 mmol), OLA (5.0 mL) and ODE (5.0 mL) were first added into a three-neck flask; then the mixture was degassed, heated to 90 °C and kept at 90 °C with the vacuum pump running for 1 h. The reaction mixture was then heated to 220 °C, and 1.0 mL of selenium/(OLA(0.7 mL) + 1-DDT(0.3 mL)) solution (1.0 M) was injected; the mixture was quickly heated to 240 °C (5 °C /min) and kept at 240 °C for 60 min. The resulting solution was mixed with 5.0 mL of hexane, and the reaction product was precipitated by adding ethanol, followed by centrifugation and re-dispersion in toluene.

### 2.3. Synthesis of ZnSe/ZnS Core/Shell NRs

In order to form core/shell ZnSe/ZnS NRs, after the growth of ZnSe NRs at 240 °C for 60 min with the presence of 1-DDT as described in the synthesis of ZnSe NRs, 3.0 mL of zinc oleate in OLA and ODE (1 M) was injected at 240 °C at the amount of 5 mL/h. The reaction mixture was kept at 240 °C for 60 min to grow ZnS shell on ZnSe NRs. It should be noted that, in this reaction, 1-DDT was employed as a sulfur source without injection of any additional S precursors.

### 2.4. Ligand Exchange on ZnSe NRs, and Fabrication of TiO_2_/ZnSe NR-Based Photoanodes

OAm and 1-DDT ligands on ZnSe NRs were partially exchanged for 3-MPA by adding 90 μL of 3-MPA to 40 mL ZnSe NRs in toluene, and adjusting the pH to 10 with 30% NaOH. After stirring for 30 min, the original ligands were partially exchanged by 3-MPA. Then, 40 mL water was added to the above solution to extract the 3-MPA-capped water-soluble ZnSe NRs. After purification by several cycles of precipitation, the 3-MPA-capped ZnSe NRs were dissolved in 1 mL water. A TiO_2_/ZnSe photoanode was made by drop casting the above ZnSe NR solution onto mesoporous TiO_2_ substrate, and keeping it for 3 h at 30 °C; then the film was rinsed by water and methanol and dried. A mesoporous TiO_2_ film was made by a doctor blading method. TiO_2_ paste was first made by mixing 0.6 g P25 TiO_2_ particles, 2.1 g alpha-terpilenol and 0.3 g ethyl cellulose using ethanol; it was doctor-bladed onto FTO glass, following by sintering at 500 °C for 30 min in air.

### 2.5. Atomic Sulfur Passivation of ZnSe NRs-Based Photoanodes

The photoanodes were dipped into a 0.1 M Na_2_S·9H_2_O solution for different durations (2, 5, 7, and 15 min) followed by rinsing with methanol and drying.

### 2.6. Depositing ZnS Layer on ZnSe NRs-Based Photoanodes by the Successive Ionic Layer Adsorption and Reaction (SILAR) Method

A TiO_2_/ZnSe photoanode was immersed into a 0.1 M Zn(NO_3_)_2_·6H_2_O solution for 1 min, then rinsed with methanol and dried. This followed by dipping into 0.1 M Na_2_S·9H_2_O solution for another 1 min to permit the formation of a ZnS monolayer. The procedure was repeated several times to control the thickness of the deposited ZnS layer. The deposition sequence has been also turned around for comparison.

### 2.7. Characterization

Transmission electron microscopy (TEM) and high-resolution TEM (HRTEM) measurements were performed on a JEOL 2100F microscope (Tokyo, Japan) at an accelerating voltage of 200 kV, with a field emission gun as the electron source. The morphology of the films was characterized by scanning electron microscope (SEM, JSM-7000, JEOL). The compositional EDX analysis and elemental mapping were carried out by EDX integrated in SEM. Powder X-ray diffraction (XRD) patterns were obtained using Cu Kα radiation on a Bruker D2 machine (Karlsruhe, Germany). Inductively coupled plasma atomic emission spectroscopy (ICP-AES) was done on PerkinElmer Optima 8000 (Waltham, MA, USA). X-ray photoelectron spectroscopy (XPS) was performed on an Thermo Fisher Scientific ESCALAB 250 (Waltham, MA, USA). Optical absorption spectra were collected on a Cary 50 spectrophotometer (Santa Clara, CA, USA). Transient photocurrent density was measured on an CHI 660 electrochemical workstation (Shanghai, China) under AM 1.5 simulated sunlight with a power density of 100 mW cm^−2^. Electrochemical impedance spectroscopy (EIS) and Mott–Schottky (MS) plot were carried out using a CHI 660 electrochemical workstation to investigate the electronic and ionic processes.

## 3. Results and Discussion

**Figure 1:**
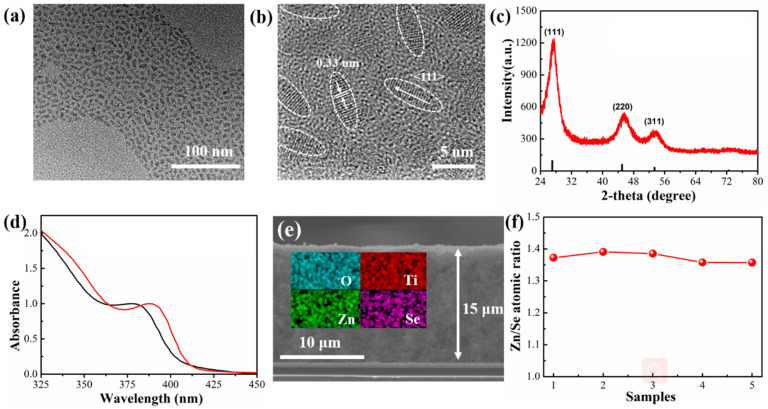
(**a**) TEM and (**b**) HRTEM images of ZnSe NRs. (**c**) XRD pattern of ZnSe NRs (in red); the line pattern in black gives the reflexes of the bulk zinc-blende ZnSe (JCPDS No. 37-1463). (**d**) UV-vis absorption spectra of ZnSe NRs before (black) and after (red) 3-MPA ligand exchange. (**e**) Cross-sectional SEM image of the TiO_2_/ZnSe NR film, with elemental distribution maps of O, Ti, Zn, and Se elements on its cross section provided as a colored inset. (**f**) Zn/Se atomic ratio for the ZnSe NRs (five samples were tested) determined by ICP–AES.

Heavy metal-free ZnSe NRs were produced via the hot-injection method with the assistance of oleyl amine (OAm) and 1-dodecanethiol (1-DDT) ligands, following a recently established synthetic procedure [42] (see Supporting Information (SI) for details). Transmission electron microscopy (TEM) images shown in Figure 1a demonstrate the rather uniform size of these ZnSe NRs. From the high resolution TEM (HRTEM) image in Figure 1b, the NRs are 2.8 ± 0.2 nm in diameter and 8.0 ± 2.0 nm in length (aspect ratio approx. 3). The marked lattice distance of 0.33 nm corresponds to (111) planes of the zinc-blende ZnSe. The cubic zinc-blende phase has been also confirmed by the X-ray diffraction (XRD) pattern provided in Figure 1c.

To apply the ZnSe NRs to PEC cells, we deposited them on a mesoporous TiO_2_ film to construct ZnSe NR-based photoanodes. A short-chain ligand of 3-MPA with bifunctional groups (–SH and –COOH) was employed to partially substitute the original long-chain ligands OLA and 1-DDT via an ex situ ligand exchange process, to facilitate charge transfer through the photoanode. The bifunctional 3-MPA ligand also served as an anchor to bind NRs to the surface of the TiO_2_ film [43]. Figure 1d shows the UV-vis absorption spectra of the ZnSe NRs before and after the ligand exchange. The bandgap of the bulk ZnSe is 2.7 eV, which corresponds to the light absorption edge of 460 nm [44,45]. The ZnSe NRs possess a diameter of ~2.8 nm, smaller than the Bohr exciton radius (3.7 nm), and thus exhibit a quantum confinement effect [46]. The first excitonic peak and the light absorption onset of the as-synthesized ZnSe NRs are thus located at 380 and 404 nm, respectively. After the ligand exchange process, both the first excitonic peak and the light absorption edge showed an 8 nm red shift. Such a red-shift likely has two possible causes. Firstly, as proposed and discussed in literature, such a red-shift can be attributed to the strong coupling between ZnSe NRs and MPA ligands [28,47,48]. Secondly, the purification of the ZnSe NRs during the ligand exchange process could also lead to a slight red-shift in the light absorption, due to the removal of surface ligands and the exposure of surface defects [49]. As already discussed in the introduction, such electron trapping states may become detrimental to the charge transfer process in PEC cells [29].

Figure S1 shows the surface morphology of the mesoporous TiO_2_/ZnSe NR photoanode. Its cross-sectional SEM image (Figure 1e) shows that the thickness of the TiO_2_ mesoporous film is approx. 15 μm. The elemental distribution maps of O, Ti, Zn, and Se on the cross section reveal the uniform distribution of ZnSe NRs on TiO_2_. Figure S2 compares the UV-vis spectra of the TiO_2_ and TiO_2_/ZnSe NR films. After immobilizing ZnSe NRs on the TiO_2_ film, the light absorption intensity was significantly enhanced, and the absorption onset extended from 380 nm to 420 nm, further confirming the successful loading of ZnSe NRs. ICP–AES was applied to determine the atomic ratio of Zn to Se in the NRs (Figure 1f), which was found to be approx. 1.4:1, despite of the same molar ratio of Zn and Se precursors (1:1) used in their synthesis. The 40% excess of Zn is thought to be surface termination atoms, which are coordinated to the ligands and under-coordinated with Se. Such a high percentage of surface termination atoms would bring the existence of charge trapping states, which may block the charge extraction and thus diminish the PEC performance [26,50].

**Figure 2:**
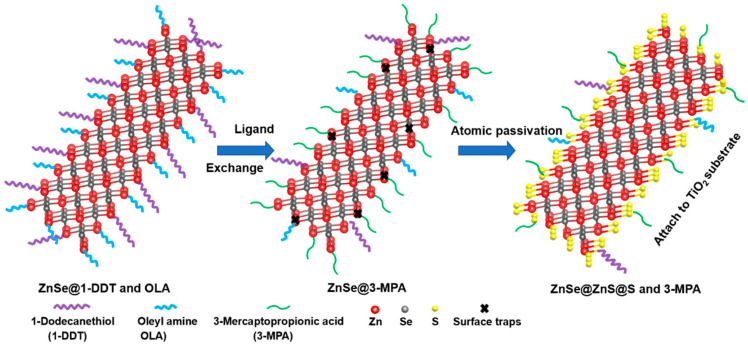
Schematic illustration of the surface configuration of ZnSe NRs during the 3-MPA ligand exchange and the subsequent atomic sulfur passivation by Na_2_S.

Aiming at passivating the surface states, we have suggested an atomic sulfide passivation method, as schematically illustrated in Figure 2. According to the previously presented data from HRTEM, XRD, and ICP–AES, ZnSe NRs consist of a stoichiometric ZnSe core with <111> orientation and a Zn-terminated surface. Before the ligand exchange, the Zn termination atoms on the surface of ZnSe NRs are coordinated with the long-chain ligands 1-DDT and OAm, which are partially replaced by the short-chain ligand 3-MPA after the ligand exchange [51,52,53]. Both the unsaturated Zn termination atoms and the surface trap states brought by ligand exchange may detrimentally influence charge transfer and lead to the accumulation of holes on the ZnSe NRs, thus resulting in their photocorrosion. To eliminate these surface states, we dipped the ZnSe NR-based photoanodes into a sodium sulfide solution to allow the coordination of S^2−^ with the surface Zn termination atoms and formation of a monolayer of ZnS. During this surface reaction, some short-chain 3-MPA ligands may become detached without sacrificing the efficient contact between the NRs and TiO_2_ film, as will be discussed later.

**Figure 3:**
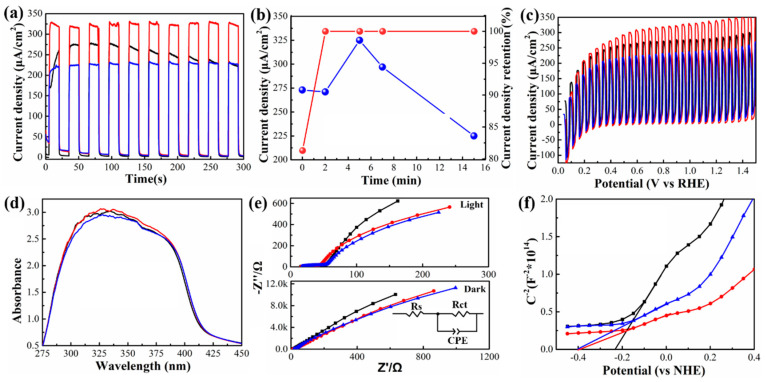
(**a**) Transient photocurrent density of TiO_2_/ZnSe NR photoanodes with atomic sulfur passivation for 0 min (black), 5 min (red), and 15 min (blue). (**b**) Dependence of the photocurrent density (blue) and photocurrent density retention (red) of TiO_2_/ZnSe NR photoanodes on the duration of the atomic sulfur passivation (data points are at 0, 2, 5, 7, and 15 min). (**c**) Light-chopped linear sweep voltammetry curves, (**d**) UV-vis absorption spectra, (**e**) electrochemical impedance spectroscopy curves under one sun illumination (AM 1.5 G) (inset: the equivalent circuit) and in the dark condition, and (**f**) Mott–Schottky plots of TiO_2_/ZnSe NR photoanodes passivated for 0 min (black), 5 min (red), and 15 min (blue). The intercepts of the Mott–Schottky plots represent the flat band potential of the photoanodes.

To evaluate the effect of the atomic sulfur passivation, we examined the transient photocurrent density of TiO_2_/ZnSe NR photoanodes produced using various passivation durations of 0, 2, 5, 7, and 15 min (denoted as TiO_2_/ZnSe/S-*x*min, where *x* represents the time of the sulfur passivation treatment) without adding any sacrificial reagents and/or co-catalysts upon light illumination (see Figure 3a and Figure S3). For the mesoporous TiO_2_ photoanode on its own, the photocurrent density was 80 μA cm^−2^ (Figure S3), which increased to 273 μA cm^−2^ after loading the ZnSe NRs (Figure 3a). However, a rather slow photo-response and poor stability of photoanodes were observed: the photocurrent density reached a peak of 275 μA cm^−2^ in almost 50 s, which lasted for less than 100 s, and then gradually decreased by 18% in only 200 s (Figure 3a). Such a slow photo-response and poor stability suggest that the charge extraction and transfer are not optimal for the non-passivated ZnSe NR-based photoanodes. Figure 3b summarizes the dependence of the photocurrent density and photocurrent density retention of TiO_2_/ZnSe NR photoanodes on the atomic sulfur passivation time. Upon increasing the atomic sulfur passivation durations, the photocurrent density underwent an increase to reach the highest value of 325 μA cm^−2^ when the passivation time was 5 min. The further increase of passivation time above 5 min resulted in a decrease in the photocurrent density even though the stability was well maintained. Finally, the photocurrent density decreased by 32.3% (220 μA cm^−2^) at the atomic sulfur passivation time of 15 min. The linear sweep voltammetry (LSV) curves of the TiO_2_/ZnSe NR photoanodes passivated for 0, 5, and 15 min are shown in Figure 3c. Compared with the sample without atomic sulfur passivation, the TiO_2_/ZnSe/S-5 min sample showed an improved photocurrent density, while the TiO_2_/ZnSe/S-15 min sample exhibited a decreased one, which further certified the optimal sulfur passivation time being 5 min.

Figure 3d illustrates the influence of the atomic sulfur passivation on the light harvesting capacity of TiO_2_/ZnSe NR photoanodes. The TiO_2_/ZnSe/S-5 min sample shows a slightly increased light absorption intensity compared with TiO_2_/ZnSe-0 min, which can be due to the formation of a ZnS monolayer on the surface [54]. We note that for the sample passivated for 15 min, the light absorption intensity decreased because of the detachment of some ZnSe NRs from the TiO_2_ surface, which could be corroborated by the data of the XPS analysis presented further below.

To obtain further insights into the effects of the atomic sulfur passivation, electrochemical impedance spectroscopy (EIS) under the illumination of an air mass 1.5 global (AM 1.5 G) solar simulator and under dark conditions was performed (see Figure 3e; an equivalent circuit model is shown in the inset). After the atomic sulfur passivation, the TiO_2_/ZnSe NR photoanodes exhibited much smaller charge transfer resistance (R_ct_), indicating a more efficient charge transfer process [55]. Figure 3f provides the Mott–Schottky plots for the TiO_2_/ZnSe NR photoanodes generated from the space charge capacitance values [56,57,58]. Positive slopes of all three curves indicate the *n*-type semiconductor characteristic of photoanodes. The flat band potentials of the photoanodes were estimated from the intercepts of the Mott–Schottky plots. Both the flat band potentials of the TiO_2_/ZnSe/S-5min and TiO_2_/ZnSe/S-15 min samples were located at ~−0.4 eV, more negative than −0.23 eV for the TiO_2_/ZnSe-0 min sample, which implies a more efficient thermodynamic driving force in the photoreduction process and a more efficient charge carrier injection.

**Figure 4:**
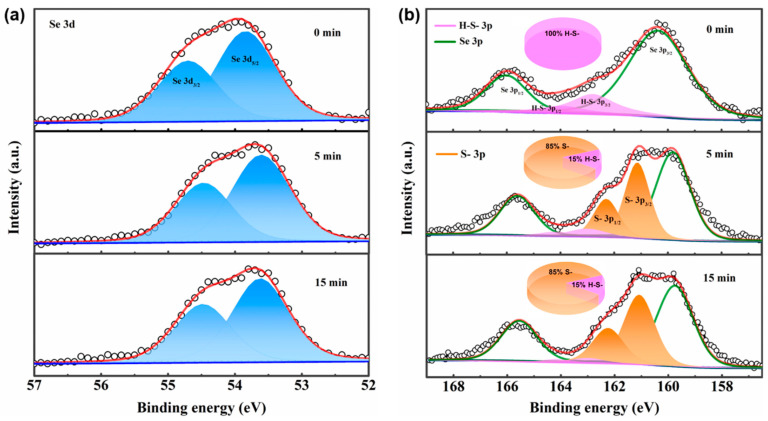
XPS spectra of (**a**) Se 3d and (**b**) S 3p of the TiO_2_/ZnSe NR photoanodes passivated with sulfur for 0, 5, and 15 min. These XPS spectra offer insights into the chemical state evolution of Se and S bonds during the atomic sulfur passivation, which were made into pie charts to demonstrate the ratios of S from the 3-MPA ligand (*H-S-*) and in ZnS (*S-*). Raw intensity of Se and S (pancyclic line graphs) and peak sums of Se and S (red lines) are also provided.

XPS analysis was conducted to reveal the evolution of chemical states of the ZnSe NRs upon atomic sulfur passivation. Figure 4a displays the high-resolution XPS spectra of Se, which could be fitted into 3d 3/2 and 3d 5/2 peaks [56]. With an increase in passivation time, the Se peaks slightly shifted to the lower energy region, and the peak position of 3d 5/2 became closer to 53.7 eV (belonging to ZnSe) [56], thus indicating a weakened surface effect on Se as a result of sulfur passivation. Figure 4b shows the high-resolution S spectra of the TiO_2_/ZnSe NR photoanodes passivated for 0, 5 and 15 min. In consideration of the fact that the S 2p peaks and Se 3p peaks are located in the same region, all XPS spectra were first fitted into two Se 3p peaks (shown by the green lines). The binding energies of the S 3p peaks can be divided into 3p 3/2 and 3p 1/2 peaks. For the TiO_2_/ZnSe-0 min photoanode, the XPS spectrum for S could be fitted by two peaks, while, for the TiO_2_/ZnSe-5 min and TiO_2_/ZnSe-15 min photoanodes, the XPS spectra for S could be represented by four peaks. The 3p 3/2 and 3p 1/2 peaks located at ~163 eV and ~164.2 eV belong to the S bond in the 3-MPA ligands (*H-S-*), while the 3p 3/2 and 3p 1/2 peaks located at ~160 eV and ~161.2 eV can be assigned to the S bond in ZnS (*S-*) [54]. By integrating the area under the fitted peaks, we found out that S originated entirely from the H-S- ligands for the TiO_2_/ZnSe-0 min photoanode without atomic sulfur passivation. For the sample passivated for 5 min, the intensity ratio of H-S-:S- became 15:85 and changed to 5:95 after a 15 min passivation. The decreased ratio of H-S-:S- upon increasing passivation time indicates that the S2^−^ ions from the sodium sulfide solution gradually exchanged some of the surface ligands and combined with the under-coordinated Zn termination atoms on the ZnSe NRs’ surface to form a ZnS monolayer. When the atomic passivation time was 15 min, the amount of the 3-MPA remaining on the surface became rather low, which inevitably weakened the linkage between the ZnSe NRs and the TiO_2_ surface, resulting in the detachment of some ZnSe NRs, which can explain the decreased light absorption intensity of the TiO_2_/ZnSe-15 min sample, as shown in Figure 3d, and the decreased photocurrent density with an increase in the atomic passivation time above 5 min. High-resolution XPS spectra of the Zn 2p region, presented in Figure S4, also shows that the peaks shifted to the lower energy region after passivation, revealing the formation of ZnS.

A comparison with other passivation methods, such as employing ZnSe/ZnS core–shell NRs, or the deposition of the ZnS layers using the SILAR method, was conducted to highlight the advantages of the atomic sulfur passivation method. ZnS shells can be directly grown on ZnSe NRs to construct core-shell ZnSe/ZnS nanorods (see Supporting Information for synthetic details), which were then deposited on mesoporous TiO_2_ films in a similar way as for ZnSe NRs. XRD patterns of ZnSe and ZnSe/ZnS NRs are compared in Figure S5a; there was no change in the peak widths, but the diffraction peak positions slightly shifted to the higher angles, which indicated the successful growth of ZnS shell [59]. A red-shift in the UV-vis absorption peak (Figure S5b) and a significant improvement in the photoluminescence intensity (Figure S5c) further proved the formation of ZnSe/ZnS core–shell NRs [60]. Figure S5d compares the transient photocurrent densities of the TiO_2_/ZnSe NR photoanodes without sulfur passivation and of the core–shell ZnSe/ZnS NR-based TiO_2_ photoanodes. The photocurrent density increased from 273 μA cm^−2^ for the former to 335 μA cm^−2^ for the latter, while the photocurrent density decreased to 242 μA cm^−2^ after 300 s.

Besides, we also applied the frequently used SILAR method in order to deposit a ZnS passivation layer on top of ZnSe NR-based TiO_2_ photoanodes (see Supporting Information for details). The transient photocurrent densities of photoanodes coated with ZnS passivation layers of different thicknesses and deposition sequences (from Zn^2+^ to S^2−^, or from S^2−^ to Zn^2+^) are shown in Figure S6a,b, respectively. With an increase in the number of SILAR deposition cycles, the photocurrent density increased and reached its maximum of 342 μA cm^−2^ after three cycles (the sequence from Zn^2+^ to S^2−^) and 375 μA cm^−2^ after six cycles (the sequence from S^2−^ to Zn^2+^). A further increase in the number of SILAR deposition cycles led to a decrease in photocurrent density, because due to the more positive maximum valence band edge of ZnS compared to that of ZnSe, the larger thickness of the ZnS layer would hinder the hole transfer from the NRs to the electrolyte [61].

**Figure 5:**
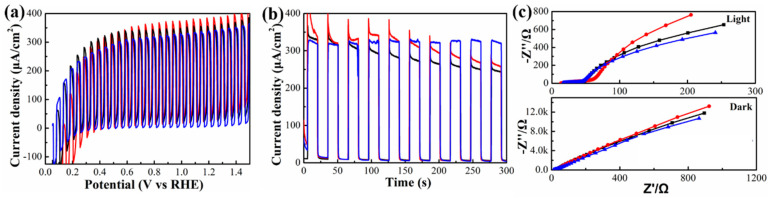
(**a**) Light-chopped LSV curves, (**b**) transient photocurrent density, and (**c**) EIS curves collected under light illumination and in the dark on the core–shell ZnSe/ZnS NR-based TiO_2_ photoanodes (black), ZnSe NR-based TiO_2_ photoanodes with post-deposited ZnS using the SILAR method (red curves), and TiO_2_/ZnSe NR photoanodes with sulfur atomic passivation for 5 mins (blue curves).

Comparisons of the LSV curves (Figure 5a) and the transient photocurrent density (Figure 5b) for photoanodes prepared by three different methods revealed that although all three passivation methods of ZnSe NRs can improve the photocurrent density of TiO_2_ photoanodes, it was only the atomic sulfur passivation method which also improved the photoanode stability at the same time. The photocurrent density of the TiO_2_/ZnSe NR photoanode passivated with atomic sulfur experienced almost no change for 300 s of the continuous operation, while the photocurrent densities of the photoanodes with ZnSe/ZnS core–shell NRs and with the post-deposited ZnS using the SILAR method declined by 28% and 25%, respectively (Figure 5b). Such an improved stability is due to the more efficient electron and hole transfer, and thus reduced photo-corrosion of the photoanodes enabled by the atomic sulfur passivation strategy, which has been possible because of several favorable aspects inherent to this particular treatment. Firstly, the surface under-coordinated Zn atoms of ZnSe NRs were efficiently coordinated by sulfur during the passivation, without causing any imperfection on the surface or the interface. Secondly, the partial replacement of 3-MPA ligands by S^2−^ during the atomic sulfur passivation may have promoted carrier mobility, enabling the faster removal of holes, and thus reducing the degree of photo-corrosion [62].

## 4. Conclusions

We have applied an atomic sulfur passivation strategy to treat the surface of ZnSe NRs used as components of TiO_2_/ZnSe photoanodes in PEC cells. As a result, without using any co-catalysts, the atomic sulfur passivation increased the photocurrent density of TiO_2_/ZnSe photoanodes from 273 to 325 μA/cm^2^. XPS study confirmed that S^2−^ ions from a sodium sulfide solution can substitute the majority of 3-MPA ligands on the ZnSe NR surface and combine with the under-coordinated Zn termination atoms to form a ZnS monolayer. Compared with ZnSe/ZnS core–shell NRs or post-depositing a ZnS passivation layer using SILAR, atomic sulfur passivation is a more efficient treatment method to facilitate the charge carrier transfer, and thus reduce the photo-corrosion of ZnSe NRs. This work highlights the advantages of the proper passivation of II-VI semiconductor nanocrystals as an efficient approach to improve the charge transfer and stability of photoelectrochemical cells based thereon.

## Supplementary Materials

The following are available online at https://www.mdpi.com/2079-4991/10/6/1081/s1: Figure S1: SEM image of the mesoporous TiO_2_/ZnSe NRs photoanode; Figure S2: UV-vis absorption spectra of the TiO_2_ photoanode (black) and the TiO_2_/ZnSe NRs photoanode without applying sulfur passivation (red); Figure S3: Transient photocurrent density of the TiO_2_ photoanode (black), and the TiO_2_/ZnSe NRs photoanodes with an atomic sulfur passivation for 2 min (red) and 7 min (blue); Figure S4: Zn 2p XPS spectra of TiO_2_/ZnSe NRs photoanodes with atomic sulfur passivation for 0, 5, and 15 min; Figure S5: (a) XRD patterns, (b) UV-vis absorption spectra, and (c) PL spectra of ZnSe NRs (black) and ZnSe/ZnS core/shell NRs (red). (d) Transient photocurrent density of TiO_2_ photoanodes modified with ZnSe NRs (black) and ZnSe/ZnS core/shell NRs (red); Figure S6: Transient photocurrent density of the TiO_2_/ZnSe NR-based photoanodes coated with ZnS passivation layers with different thickness and deposition sequence, (a) from Zn^2+^ to S^2−^ and (b) from S2^−^ to Zn^2+^ using successive ionic layer adsorption and reaction (SILAR) method.
