# Supplementary material for: Atomic Sulfur Passivation Improves the Photoelectrochemical Performance of ZnSe Nanorods
Source: Nanomaterials (Basel). 2020 May 31;10(6):1081. doi: 10.3390/nano10061081 (PMC7353383; doi:10.3390/nano10061081)
Supplement: Supplementary file 1 [file nanomaterials-10-01081-s001.pdf]

Supporting Information

Atomic Sulfur Passivation Improves the Photoelectrochemical Performance of ZnSe Nanorods

Fei Huang [1,2,3,*,†], Jiajia Ning [3,4,†], Wei Xiong [2,3], Ting Shen [1], Yanling Zhao [2], Jianjun Tian [1], Ruiqin Zhang [2,3] and Andrey L. Rogach [3,4]

[1] Institute for Advanced Materials and Technology, University of Science and Technology Beijing, Beijing 100083, China; ting_shen@sutd.edu.sg (T.S.); tianjianjun@mater.ustb.edu.cn (J.T.)

[2] Department of Physics, City University of Hong Kong, 999077, Hong Kong, China; weixiong4-c@my.cityu.edu.hk (W.X.); apzyl@cityu.edu.hk (Y.Z.); aprqz@cityu.edu.hk (R.Z.)

[3] Center for Functional Photonics (CFP), City University of Hong Kong, 999077, Hong Kong, China; jiajning@cityu.edu.hk (J.N.); andrey.rogach@cityu.edu.hk (A.L.R.)

[4] Department of Material Science and Engineering, City University of Hong Kong, 999077, Hong Kong, China

* Correspondence: huangfei@ustb.edu.cn

† These authors contributed equally to this work.

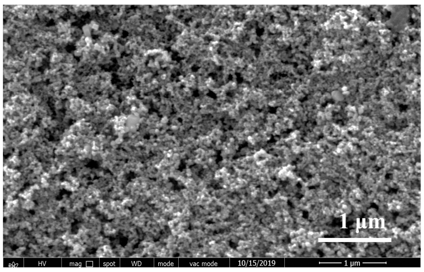

Figure S1. SEM image of the mesoporous TiO_2/ZnSe NRs photoanode.

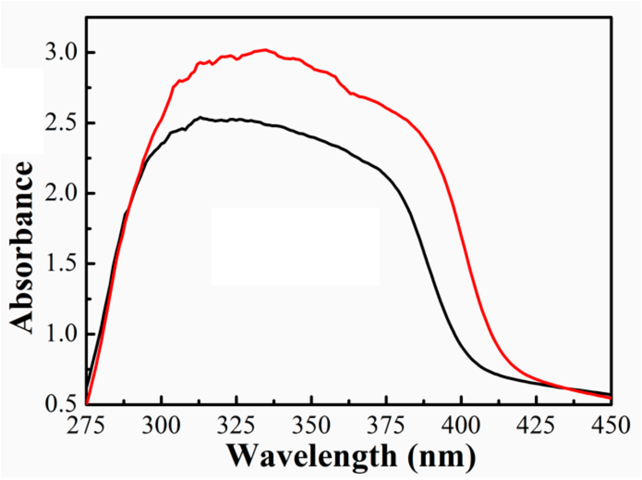

Figure S2. UV-vis absorption spectra of the TiO_2 photoanode (black) and the TiO_2/ZnSe NRs photoanode without applying sulfur passivation (red).

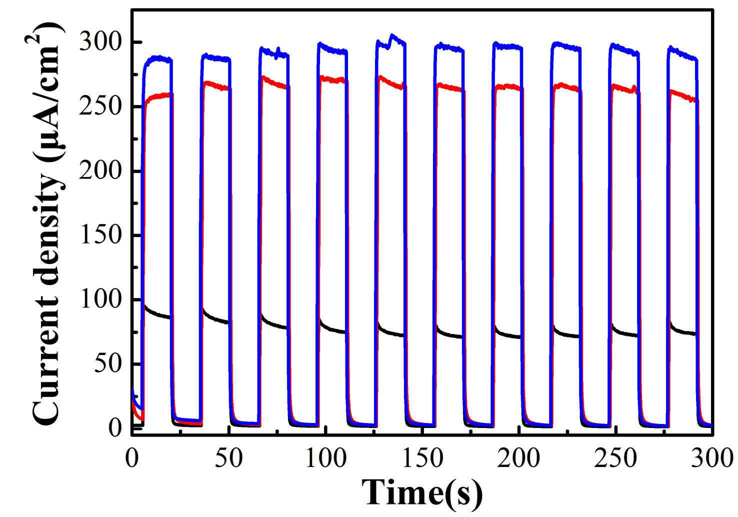

Figure S3. Transient photocurrent density of the TiO_2 photoanode (black), and the TiO_2/ZnSe NRs photoanodes with an atomic sulfur passivation for 2 min (red) and 7 min (blue).

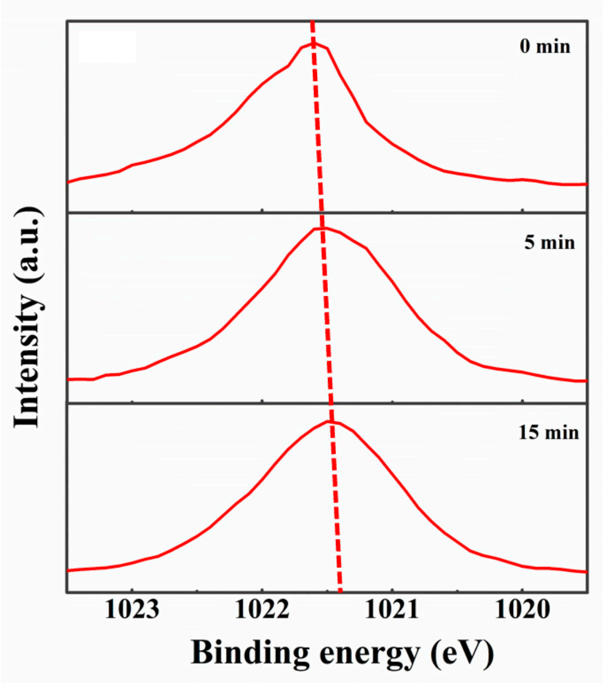

Figure S4. Zn 2p XPS spectra of TiO_2/ZnSe NRs photoanodes with atomic sulfur passivation for 0, 5, and 15 min.

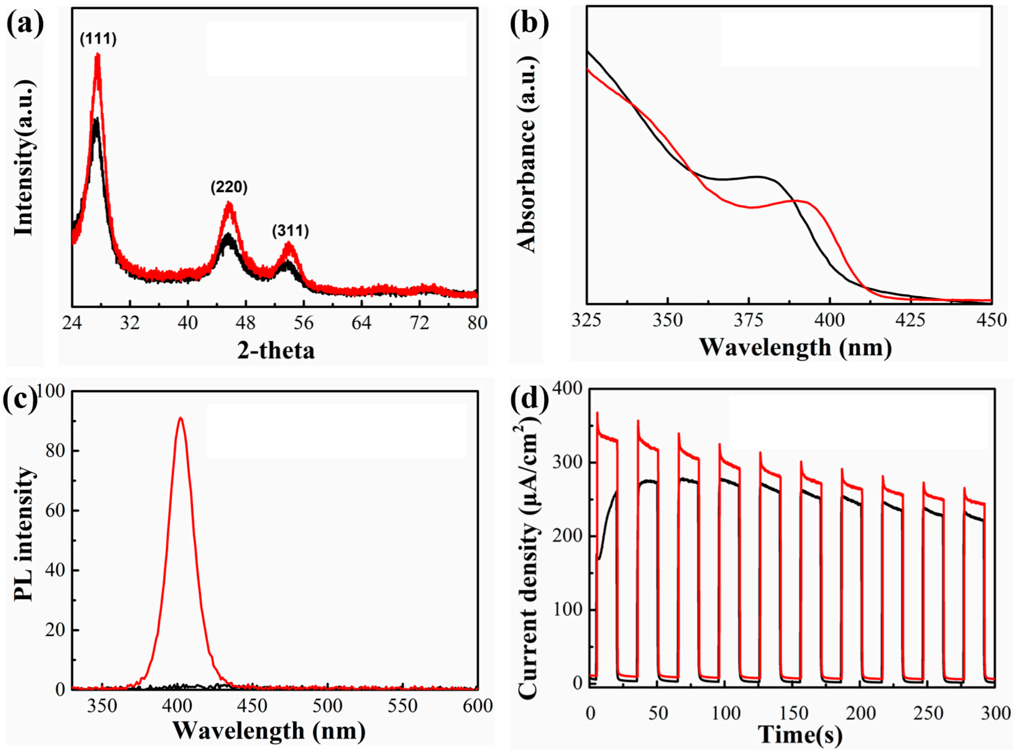

Figure S5. (**a**) XRD patterns, (**b**) UV-vis absorption spectra, and (**c**) PL spectra of ZnSe NRs (black) and ZnSe/ZnS core/shell NRs (red). (**d**) Transient photocurrent density of TiO_2 photoanodes modified with ZnSe NRs (black) and ZnSe/ZnS core/shell NRs (red).

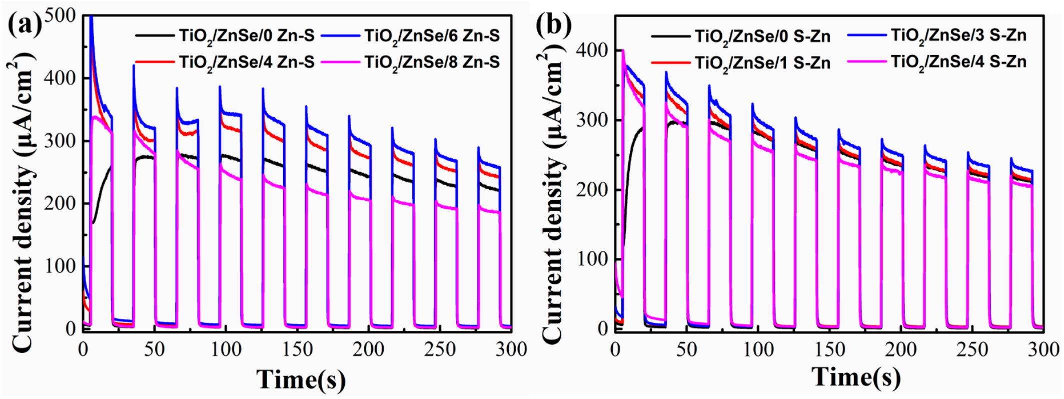

Figure S6. Transient photocurrent density of the TiO_2/ZnSe NR-based photoanodes coated with ZnS passivation layers with different thickness and deposition sequence, (**a**) from Zn^{2+} to S^{2-} and (**b**) from S^{2-} to Zn^{2+} using successive ionic layer adsorption and reaction (SILAR) method.
